# Supplementary material for: Revisiting historical beech and oak forests in Indiana using a GIS method to recover information from bar charts
Source: PeerJ. 2018 Jul 6;6:e5158. doi: 10.7717/peerj.5158 (PMC6037136; doi:10.7717/peerj.5158)
Supplement: Figure S1 [file peerj-06-5158-s001.pdf]

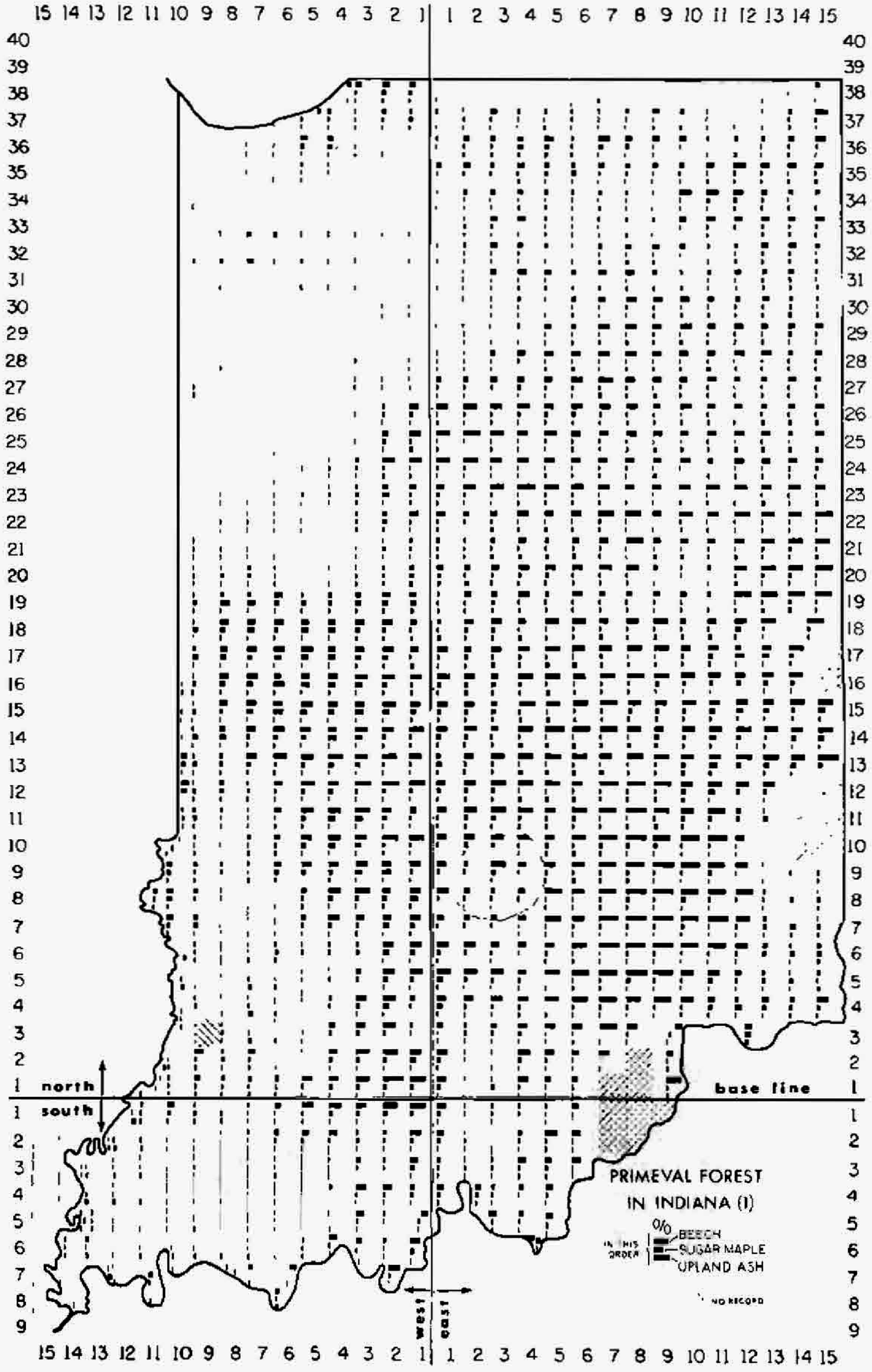

Fig. 1. Map-graph showing distribution in Indiana of the climax beech-sugar maple-upland ash forest association as percentages of total witness trees recorded by townships in the original U. S. Land Survey. (Copies of the tables of percenrages on which this and figures 2 and 3 are based bave hcen deposited in the Butler University Library, Indianapolis, Indiana.)
